# Cadmium Exposure Modulates Quality of the Medicinal Plant *Dendrobium officinale* via Regulation of Gene Expression, Antioxidant Systems, Bioactive Compound Accumulation, and Rhizosphere Microbial Community

**DOI:** 10.3390/plants15203187

**Published:** 2026-10-17

**Authors:** Hexigeduleng Bao, Yu Wang, Hainan Bao, Feijuan Wang, Qiong Jiang, Hua Li, Yanfei Ding, Cheng Zhu

**Affiliations:** 1 Key Laboratory of Specialty Agri-Product Quality and Hazard Controlling Technology of Zhejiang Province, College of Life Science, China Jiliang University, Hangzhou 310018, Chinaqjiang@cjlu.edu.cn (Q.J.);; 2 College of Smart Agriculture (College of Artificial Intelligence), Nanjing Agricultural University, Nanjing 210031, China

**Keywords:** heavy metal pollution, cadmium, *Dendrobium officinale*, medicinal plants, bioactive compounds, rhizosphere microbial community

## Abstract

With increasing industrialization, heavy metal contamination poses a growing threat to ecosystem stability and human health. Cadmium (Cd) is taken up by medicinal plants through the soil–plant continuum, altering rhizosphere microbial communities and thereby affecting plant metabolism and bioactive compound accumulation. In this study, *Dendrobium officinale* Kimura & Migo (*D. officinale*) was used as a model medicinal plant to investigate the effects of Cd exposure on plant quality and rhizosphere microbial community composition. The results show that different *D. officinale* strains exhibited significant differences in growth traits and Cd accumulation under Cd exposure. Under the 0.9 mg·kg^−1^ Cd treatment, mean Cd concentrations in the strain with the highest Cd accumulation (Strain 2) reached 6.42, 1.08, and 1.70 mg·kg^−1^ DW in roots, stems, and leaves, respectively, compared with 2.13, 0.73, and 0.66 mg·kg^−1^ DW in the strain with the lowest Cd accumulation (Strain 1), corresponding to approximately 3.0-, 1.5-, and 2.6-fold higher accumulation. Cd exposure was associated with strain-specific changes in metal transport-related gene expression, antioxidant responses, bioactive constituent accumulation, and rhizosphere microbial community composition. Together, these changes highlight the complex relationship between Cd accumulation, plant physiological responses, and medicinal quality. These findings provide a theoretical reference for the selection of elite *D. officinale* strains and for the safe cultivation of this medicinal plant.

## 1. Introduction

Increasing industrialization and agricultural intensification have made heavy metal contamination a major global environmental challenge. Cadmium (Cd) is a highly toxic and mobile heavy metal that can accumulate in agricultural soils and subsequently enter plants [1]. The extent of Cd accumulation can vary substantially among plant genotypes, resulting in genotype-dependent differences in tissue Cd concentrations and production safety [2]. Medicinal plants are particularly vulnerable because their quality directly affects the safety and therapeutic efficacy of herbal products. When medicinal plants are cultivated in contaminated environments for extended periods, heavy metals can accumulate in plant tissues and remain in harvested materials [3], while also disrupting plant metabolism and reducing bioactive compound accumulation [4,5], thereby posing potential risks to human health. Heavy metal contamination not only affects the quality of medicinal plants but also influences plant metabolism by altering the composition and function of rhizosphere microbial communities [6]. In this context, plant responses to Cd exposure are governed not only by plant physiological regulation but also by interactions within the rhizosphere environment. The rhizosphere of medicinal plants harbors diverse rhizosphere microbial communities, including bacteria, fungi, and actinomycetes [7], which establish complex and dynamic interactions with plant roots, forming structured ecological networks linking plants and soil [8]. Under Cd exposure, soil microorganisms play pivotal roles in plant adaptation by modulating metal speciation, altering bioavailability, and participating in stress-response pathways [9]. Heavy-metal contamination can alter the assembly patterns and key taxa of rhizosphere bacterial communities, indicating that rhizosphere microbial restructuring represents an important component of plant responses to Cd-contaminated environments [10].

*D. officinale*, a traditionally valued medicinal herb in China, is recognized as one of the “Nine Great Immortal Herbs” [11]. As a representative medicinal plant, it has important medicinal and clinical value. Modern pharmacological and clinical studies have demonstrated that *D. officinale* contains diverse bioactive compounds, including polysaccharides, flavonoids, alkaloids, and dendrophenol [12], which are associated with multiple pharmacological activities, including hypoglycemic [13], antitumor [14], antioxidant [15], anti-inflammatory [16], and immunomodulatory effects [17]. With increasing demand for medicinal and food products, the cultivation of *D. officinale* has expanded rapidly in China, and artificial cultivation has become the dominant production mode. Recent reports indicate that *D. officinale* has been artificially cultivated on a large scale in southern China, particularly in Zhejiang, Yunnan, Anhui, Guangdong, and Guangxi provinces [18].

However, *D. officinale* typically grows on gravelly substrates or under cultivated conditions, making it susceptible to Cd contamination, which adversely affects plant growth and medicinal plant quality. Notably, different strains of *D. officinale* exhibit considerable differences in Cd tolerance and bioactive compound accumulation, and these genetic differences are considered important determinants of quality formation [19]. With the expansion of large-scale cultivation, cultivation environments have become increasingly heterogeneous, increasing the likelihood of Cd exposure during production [20]. Previous studies have demonstrated that Cd exposure can induce oxidative stress, alter Cd subcellular distribution, and substantially reprogram transcriptional and metabolic processes in *D. officinale*. In particular, Cd stress has been associated with changes in antioxidant responses, glutathione metabolism, cell-wall metabolism, and phenylpropanoid pathways, indicating that Cd exposure may simultaneously affect plant growth, stress tolerance, and the metabolic basis of medicinal quality [21]. Moreover, monitoring studies have detected Cd in cultivated *D. officinale* materials, and Cd accumulation can vary among germplasms and physiological ages, underscoring the importance of controlling heavy-metal contamination during production and quality assessment [22]. Although previous studies have established that Cd exposure and rhizosphere microbial communities significantly influence plant metabolism and physiological characteristics of medicinal plants [23], the relationship between Cd-induced changes in medicinal plant quality and the composition and dynamics of rhizosphere microbial communities in *D. officinale* remains largely unclear. Moreover, differences among distinct *D. officinale* strains in their response to Cd exposure and associated shifts in rhizosphere microbial communities remain to be elucidated.

To address these gaps, this study aimed to investigate the interactions among Cd exposure, *D. officinale*, and rhizosphere microbial communities, and to systematically evaluate the effects of Cd exposure on the medicinal plant quality of different *D. officinale* strains and their underlying mechanisms. Changes in growth traits following Cd exposure were evaluated, together with Cd accumulation in different tissues, particularly medicinal tissues. In addition, physiological characteristics related to oxidative stress were analyzed to assess Cd-induced oxidative damage and antioxidant responses. The expression of metal ion transport-related genes was further examined, and the accumulation of bioactive compounds, including polysaccharides, flavonoids, alkaloids, anthocyanins, and soluble protein, was determined to evaluate how Cd exposure affects quality formation in *D. officinale*. High-throughput sequencing was also performed to characterize changes in the diversity and composition of rhizosphere microbial communities under Cd exposure, thereby exploring potential associations between microbial community shifts and medicinal plant quality.

By integrating data on growth traits, physiological characteristics, gene expression, bioactive compound accumulation, and rhizosphere microbial communities, this study aimed to elucidate the effects of Cd exposure on the medicinal plant quality of *D. officinale* and to identify potential associations between shifts in rhizosphere microbial communities and quality formation. These findings provide a scientific basis for the safe cultivation of *D. officinale* and contribute to the risk assessment and management of heavy metal contamination.

## 2. Results

### 2.1. Growth Traits and Cd Accumulation Under Cd Treatment

**Figure 1:**
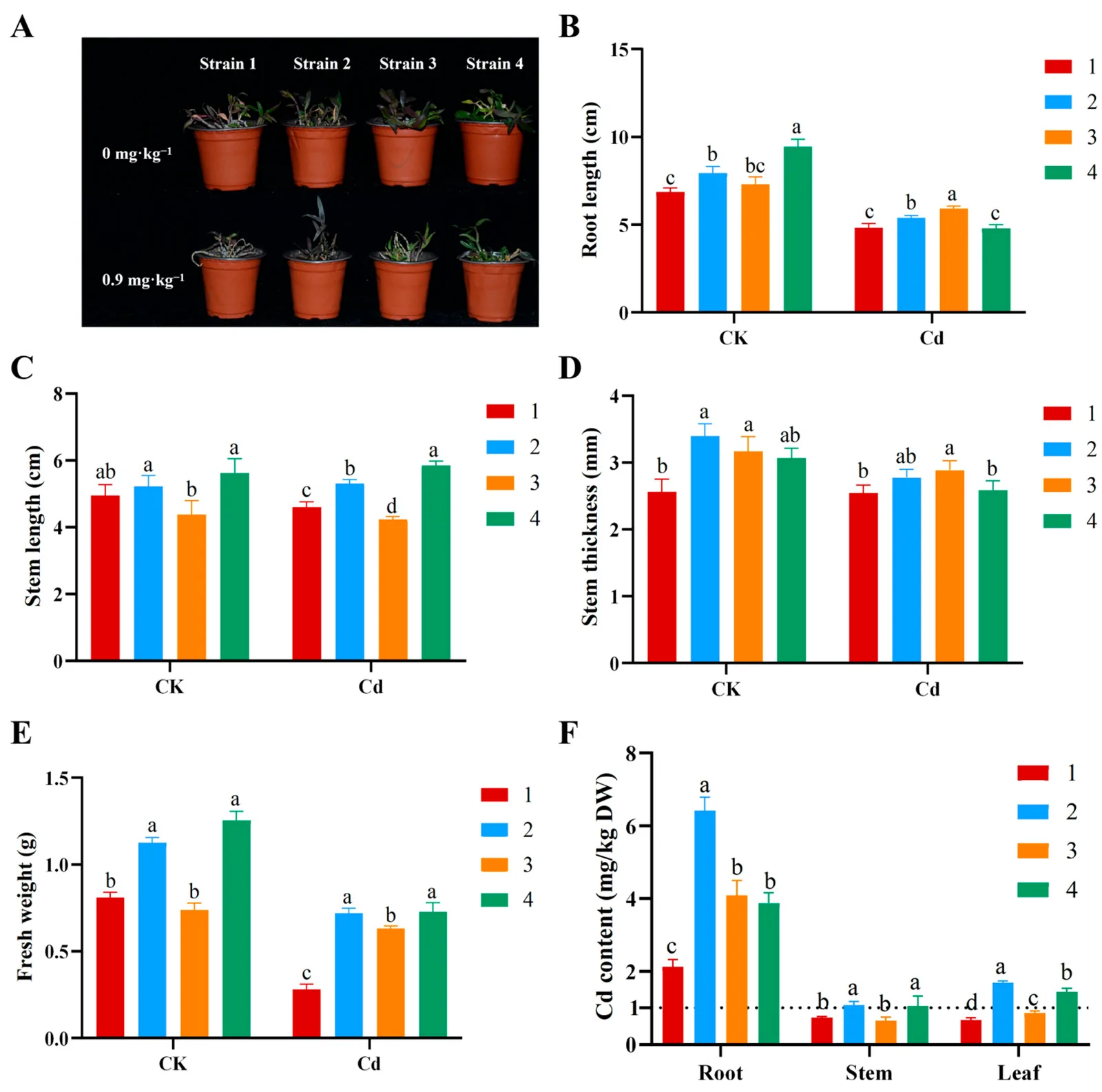
Phenotypic responses, growth traits, and Cd accumulation in different *D. officinale* strains under CK and Cd treatments. (**A**) Representative phenotypes of the four strains under 0 and 0.9 mg·kg^−1^ Cd treatments. (**B**) Root length. (**C**) Stem length. (**D**) Stem thickness. (**E**) Fresh weight. (**F**) Cd concentrations in roots, stems, and leaves of each strain. For panels (**B**–**F**), CK represents the 0 mg·kg^−1^ Cd treatment, whereas Cd represents the 0.9 mg·kg^−1^ Cd treatment. The dotted horizontal line in panel (**F**) indicates the maximum Cd limit of 1 mg·kg^−1^ for medicinal tissues specified by the Chinese Pharmacopoeia. Data are presented as mean ± SD (*n* = 10). Different lowercase letters indicate significant differences among strains within the same treatment or tissue (*p* < 0.05). Strains are numbered as follows: 1, Strain 1 (Taizhou strain); 2, Strain 2 (Xianju strain); 3, Strain 3 (Fujian strain); and 4, Strain 4 (Wenzhou strain).

After one month of Cd treatment, the phenotypic responses of the four *D. officinale* strains under different Cd concentrations are shown in Figure 1A. Marked phenotypic differences were observed among the strains under Cd exposure. In the control (0 mg·kg^−1^ Cd), all strains exhibited generally healthy growth. Under 0.9 mg·kg^−1^ Cd treatment, plants showed pronounced wilting and growth inhibition. Among the four strains, Strain 1 exhibited the most severe visible injury symptoms.

For quantitative comparison of growth traits, two representative treatments (0 mg·kg^−1^, CK treatment; and 0.9 mg·kg^−1^, Cd treatment) were selected for comparative analysis. Growth traits for each strain are presented in Figure 1B–E. Under CK conditions, all *D. officinale* strains showed normal growth. In contrast, Cd treatment reduced growth traits to different extents, with clear strain-dependent differences. Cd treatment affected the four growth traits in a strain- and trait-dependent manner. Root length decreased in all four strains, with the greatest reduction observed in Strain 4 (approximately 49.2%) and the smallest reduction in Strain 3 (approximately 19.0%). Stem length decreased slightly in Strains 1 and 3 but remained stable or slightly increased in Strains 2 and 4. Stem thickness showed little change in Strain 1, whereas larger reductions were observed in Strains 2 and 4. Fresh weight decreased in all strains, with the greatest reduction in Strain 1 (approximately 65.4%) and the smallest reduction in Strain 3 (approximately 14.4%). These quantitative growth responses were generally consistent with the visible phenotypic changes observed in Figure 1A.

As shown in Figure 1F, Cd concentrations in the roots of all four strains exceeded 2 mg·kg^−1^. In the medicinal tissues (stems and leaves), Cd concentrations in Strains 2 and 4 exceeded the maximum limit of 1 mg·kg^−1^ specified by the Chinese Pharmacopoeia, whereas those in Strains 1 and 3 remained below this threshold. Strain 2 exhibited the highest Cd concentrations in the roots, stems, and leaves (6.42, 1.08, and 1.70 mg·kg^−1^ DW, respectively) among all strains. In contrast, Strain 1 showed lower Cd concentrations across all tissues (2.13, 0.73, and 0.66 mg·kg^−1^ DW in roots, stems, and leaves, respectively).

The bioconcentration factor (BCF) is used to assess the ability of a plant to absorb and accumulate a specific element, particularly heavy metals or pollutants, from the soil [24]. It is calculated as BCF = C_sample_/C_soil_. The translocation factor (TF) evaluates the relative distribution of Cd from roots to aboveground tissues and was calculated as TF = C_aboveground_/C_root_, where C_aboveground_ represents the Cd concentration in the aboveground part and C_root_ represents the Cd concentration in roots [24]. As shown in Table 1, based on the original Cd concentration data, the recalculated TF values for Strains 1–4 were 0.656, 0.433, 0.370, and 0.644, respectively. Strain 1 exhibited the lowest BCF value, indicating relatively low overall Cd accumulation. All four strains had TF values below 1, showing that Cd concentrations were predominantly higher in roots than in aboveground tissues. Strain 2 exhibited a relatively low TF (0.433) but the highest absolute Cd concentrations in roots, stems, and leaves. Therefore, its relatively high Cd concentrations in aboveground tissues were associated primarily with its greater overall Cd accumulation.

### 2.2. Changes in Physiological Characteristics

**Figure 2:**
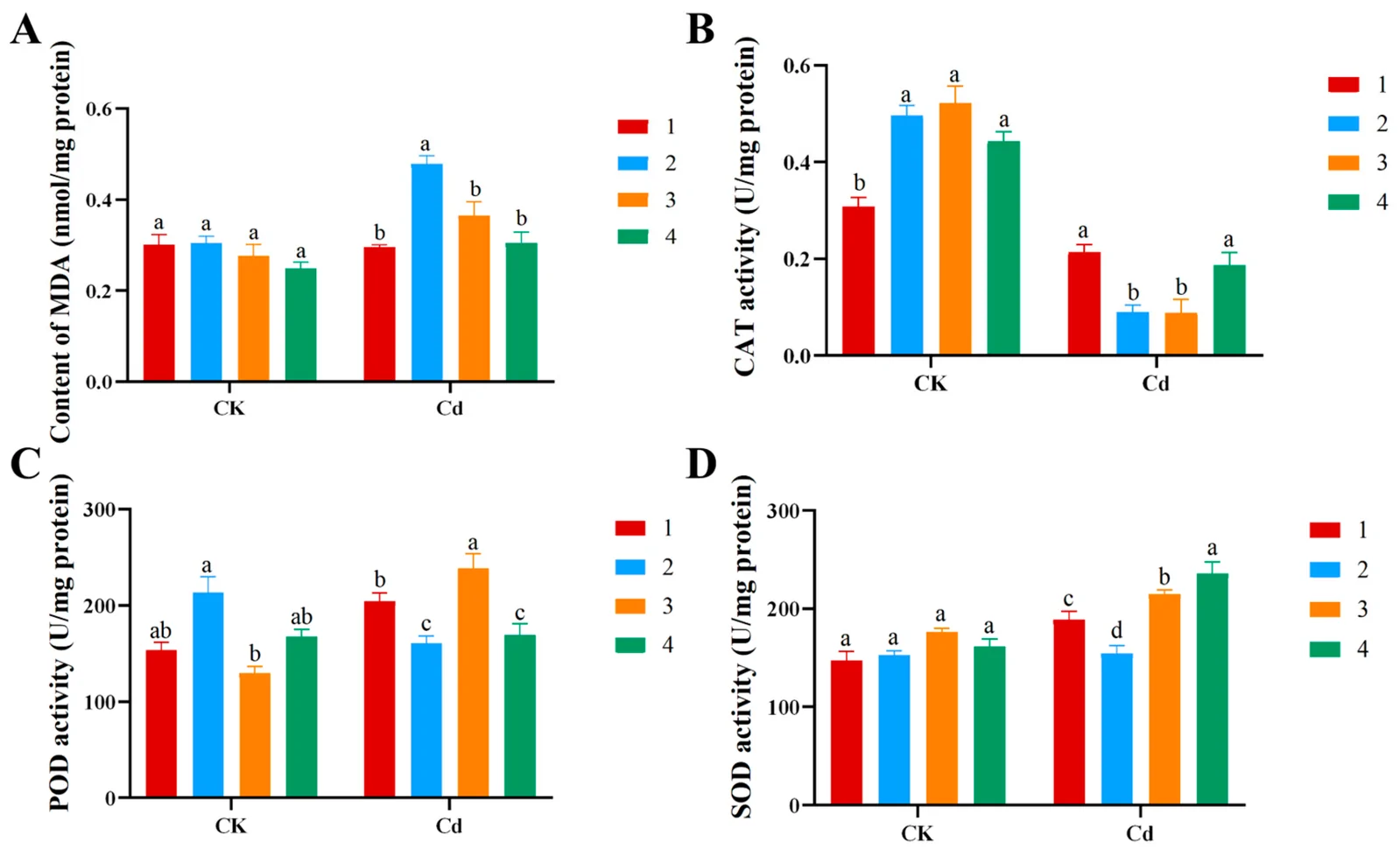
Changes in physiological characteristics in different *D. officinale* strains under Cd treatment. (**A**) MDA content; (**B**) CAT activity; (**C**) POD activity; (**D**) SOD activity. Cd treatment altered lipid peroxidation and antioxidant enzyme activities to different extents among the four strains. MDA content increased in Strains 2, 3, and 4 but changed little in Strain 1; CAT activity decreased in all strains, whereas POD and SOD activities showed strain-dependent responses. CK represents the 0 mg·kg^−1^ Cd treatment, whereas Cd represents the 0.9 mg·kg^−1^ Cd treatment. Data are presented as mean ± SD (*n* = 3). Different lowercase letters indicate significant differences among strains within the same treatment or tissue (*p* < 0.05). Strains are numbered as follows: 1, Strain 1 (Taizhou strain); 2, Strain 2 (Xianju strain); 3, Strain 3 (Fujian strain); and 4, Strain 4 (Wenzhou strain).

After Cd treatment, the physiological characteristics of the four *D. officinale* strains showed marked differences (Figure 2). Malondialdehyde (MDA) content increased markedly in Strains 2, 3, and 4, particularly in Strain 2, whereas a slight decrease was observed in Strain 1. Cd-induced MDA accumulation has also been reported in *D. officinale*, reflecting Cd-associated oxidative stress [21]. Activities of catalase (CAT) activity decreased in all four strains, with the greatest reductions in Strains 2 and 3. Because CAT is an important component of the antioxidant system involved in H_2_O_2_ detoxification under Cd stress, reduced CAT activity may weaken H_2_O_2_-scavenging capacity [25]. Peroxidase (POD) activity increased in Strains 1 and 3, decreased in Strain 2, and changed only slightly in Strain 4, suggesting activation of antioxidant defense mechanisms to alleviate Cd-induced oxidative damage through enhanced H_2_O_2_ scavenging [26]. Superoxide dismutase (SOD) activity increased in Strains 1, 3, and 4, indicating a strain-dependent enhancement of antioxidant defense in response to Cd exposure. Antioxidant regulation has previously been reported as an important component of the Cd response in *D. officinale* [21]. These findings demonstrate strain-specific patterns of lipid peroxidation and antioxidant-enzyme regulation under Cd exposure.

### 2.3. Cd-Related Gene Expression Changes

**Figure 3:**
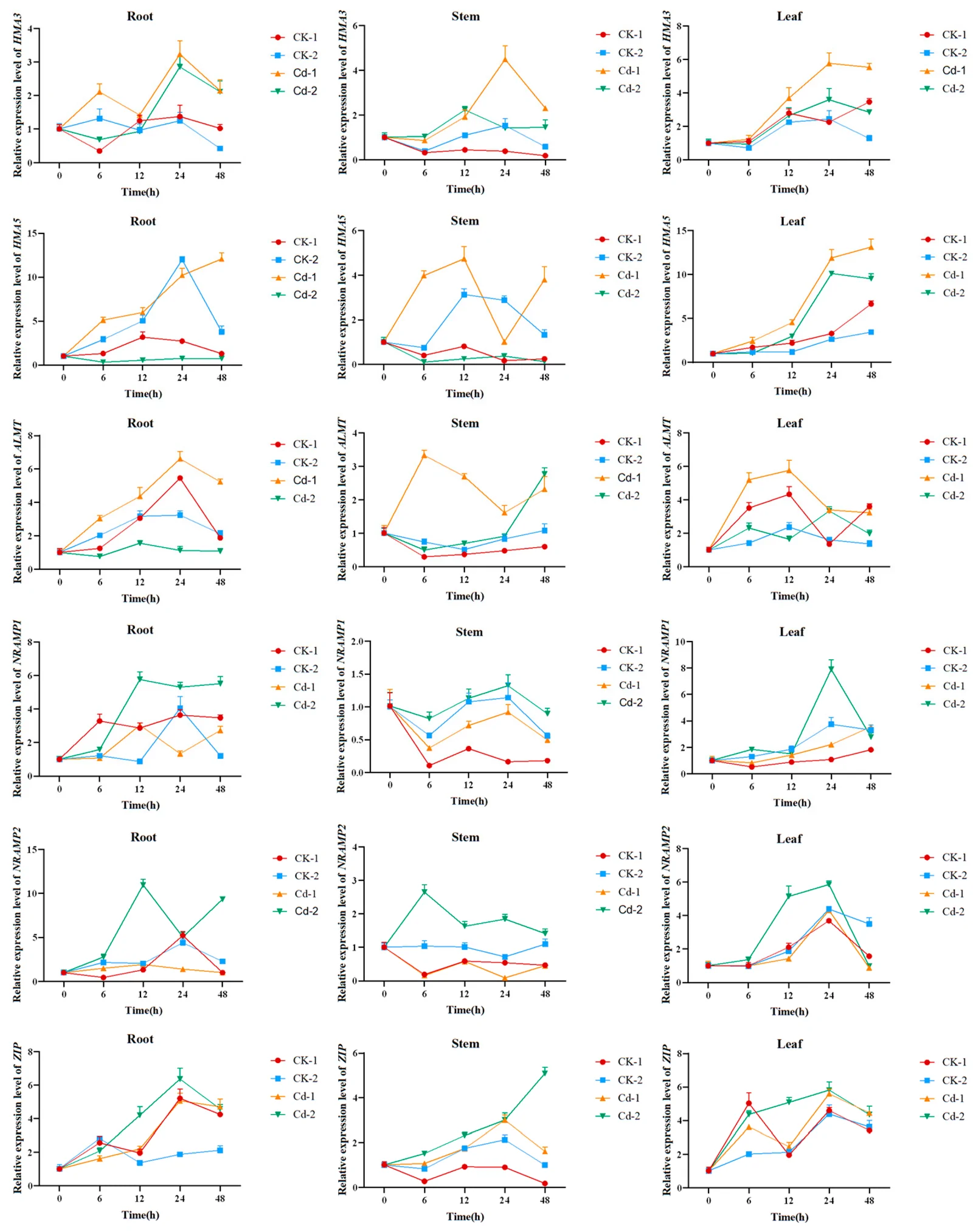
Expression profiles of Cd-related genes in different tissues of *D. officinale* strains under short-term Cd treatment. Relative expression levels of *HMA3*, *HMA5*, *ALMT*, *NRAMP1*, *NRAMP2*, and *ZIP* were determined in roots, stems, and leaves at 0, 6, 12, 24, and 48 h after treatment. Cd treatment rapidly induced the upregulation of genes associated with heavy metal transport regulation, including *HMA3*, *HMA5*, and *ALMT*, in Strain 1. In contrast, Strain 2 showed stronger early upregulation of genes involved in heavy metal transport, including *NRAMP1*, *NRAMP2*, and *ZIP*. Data are presented as mean ± SD (*n* = 3). Strains were designated as follows: CK-1, Strain 1 (Taizhou strain) under CK treatment; CK-2, Strain 2 (Xianju strain) under CK treatment; Cd-1, Strain 1 (Taizhou strain) under Cd treatment; Cd-2, Strain 2 (Xianju strain) under Cd treatment.

Based on the above results, Strains 1 and 2, which showed distinct differences, were selected for further investigation. Under short-term Cd treatment, the expression patterns of key heavy metal-related genes, including *HMA3/5*, *ALMT*, *NRAMP1/2*, and *ZIP*, showed clear strain- and organ-dependent expression patterns between the two strains (Figure 3). Most pronounced transcriptional changes were observed within 6–24 h after Cd treatment, consistent with previous time-course transcriptomic studies showing rapid Cd-responsive transcriptional reprogramming and transporter regulation during the first 6–24 h of exposure [27]. In Strain 1, the expression of *HMA5* and *ALMT* showed stronger and more sustained upregulation following Cd treatment, with distinct peaks at 12–24 h. The sustained induction of *HMA5* suggests that this transporter may participate in Cd-associated metal transport and redistribution. *HMA5* homologs have been implicated in Cd transport in other plant species; however, the specific direction and physiological consequence of *HMA5*-mediated transport in *D. officinale* require further functional validation [28]. Concurrently, the upregulation of *ALMT* may promote organic acid-mediated Cd chelation or cell wall/vacuolar sequestration [29], thereby further reducing Cd toxicity and translocation to aboveground tissues. In contrast, Strain 2 exhibited stronger induction of several genes, particularly *NRAMP1*, *NRAMP2*, and *ZIP*, following Cd treatment, with peak expression occurring around 24 h. These genes are predominantly involved in the transmembrane uptake and long-distance transport of divalent metals [30,31]. The temporal and tissue-specific expression patterns of metal transport-related genes indicate differential regulation of metal homeostasis between the two strains, which may contribute to explaining the higher Cd concentrations observed in the aboveground tissues of this strain.

**Figure 4:**
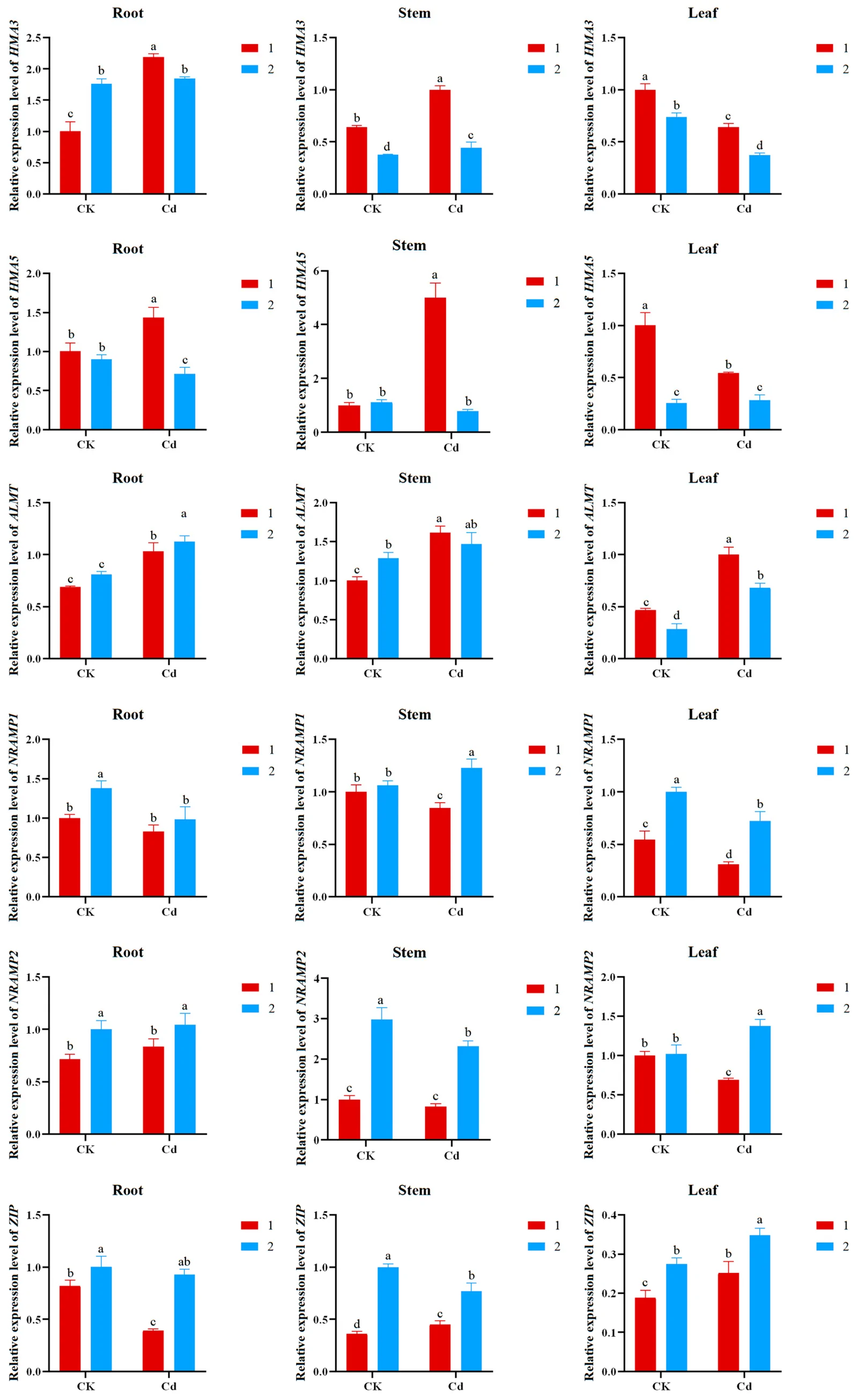
Expression profiles of Cd-related genes in different tissues of *D. officinale* strains after one month of Cd treatment. The three columns represent roots, stems, and leaves, respectively, and the six rows show the relative expression levels of *HMA3*, *HMA5*, *ALMT*, *NRAMP1*, *NRAMP2*, and *ZIP*. In Strain 1, *HMA3* and *HMA5* increased mainly in roots and stems but decreased in leaves, whereas *ALMT* increased across the three tissues, with increased expression of genes associated with metal sequestration and efflux. In Strain 2, *NRAMP1*, *NRAMP2*, and *ZIP* displayed tissue-dependent changes rather than uniform upregulation across roots, stems, and leaves, with increased expression of genes associated with metal uptake and translocation to aboveground tissues. Data are presented as mean ± SD (*n* = 3). Different lowercase letters indicate significant differences among strains within the same treatment or tissue (*p* < 0.05). Strains were designated as follows: 1, Strain 1 (Taizhou strain); 2, Strain 2 (Xianju strain).

After one month of Cd treatment, the two strains showed distinct differences in the expression levels of heavy metal transport-related genes (Figure 4). In Strain 1, *HMA3* expression increased in roots and stems but decreased in leaves after one month of Cd treatment. Similarly, *HMA5* expression increased in roots and stems, with a particularly strong increase in stems, but decreased in leaves. Concurrently, *ALMT* was significantly induced in all three organs of this strain, suggesting that Strain 1 may respond to Cd stress through organic acid-mediated chelation and detoxification processes [32]. In Strain 2, *NRAMP1*, *NRAMP2*, and *ZIP* showed distinct tissue-specific responses rather than uniform upregulation. *NRAMP1* increased in stems but decreased in roots and leaves. *NRAMP2* increased in leaves, remained relatively stable in roots, and decreased in stems. *ZIP* increased in leaves, remained relatively stable in roots, and decreased in stems. These results suggest that Cd exposure induced distinct tissue-specific transcriptional responses in the two strains, which may contribute to their different Cd accumulation patterns.

### 2.4. Changes in Bioactive Constituent Contents

**Figure 5:**
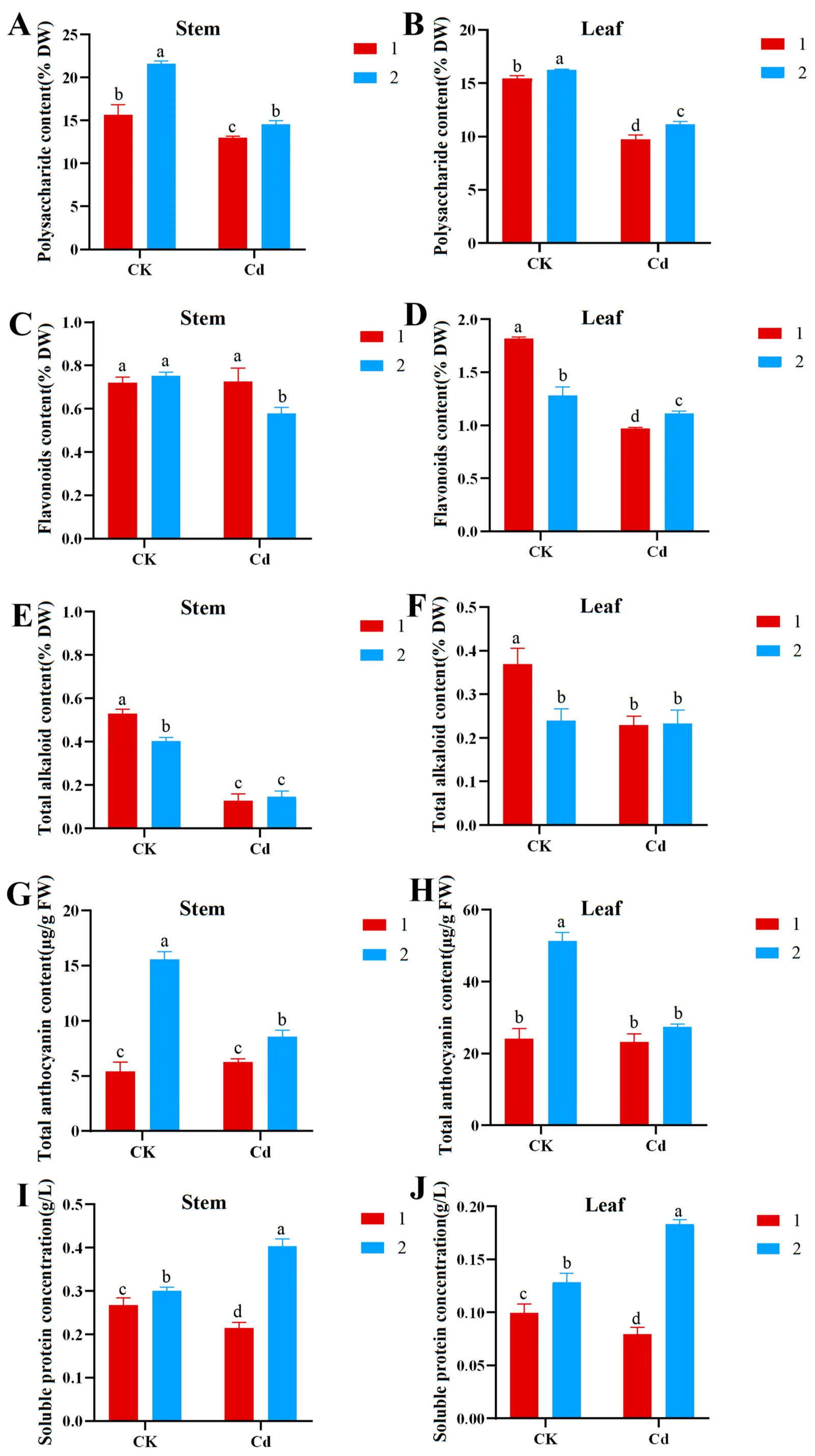
Changes in bioactive constituent contents in stems and leaves of different *D. officinale* strains under different treatments. (**A**,**B**) Polysaccharides. (**C**,**D**) Flavonoids. (**E**,**F**) Alkaloids. (**G**,**H**) Anthocyanins. (**I**,**J**) Soluble protein. Panels (**A**,**C**,**E**,**G**,**I**) show the stem samples, whereas panels (**B**,**D**,**F**,**H**,**J**) show the leaf samples. Cd treatment generally reduced the contents of polysaccharides, flavonoids, and alkaloids. Anthocyanin contents generally decreased in the stems and leaves of both strains. In contrast, soluble protein content increased significantly in Strain 2, and bioactive constituent contents in Strain 2 remained more stable under Cd treatment. Data are presented as mean ± SD (*n* = 3). Different lowercase letters indicate significant differences among strains within the same treatment or tissue (*p* < 0.05). Strains are numbered as follows: 1, Strain 1 (Taizhou strain); 2, Strain 2 (Xianju strain).

The changes in polysaccharide, flavonoid, and alkaloid contents in the stems and leaves of the two strains under different treatments are presented in Figure 5. As shown in Figure 5A,B, compared with the CK group, Cd treatment resulted in decreased polysaccharide contents in both stems and leaves of both strains. However, Strain 2 showed higher polysaccharide contents in both tissues than Strain 1. Regarding flavonoid content (Figure 5C,D), under CK treatment, no significant difference was observed between the two strains in stems, whereas Strain 1 showed higher flavonoid content in leaves than Strain 2. After Cd treatment, flavonoid content decreased to varying extents in both stems and leaves of both strains, with the greatest decrease observed in the leaves of Strain 1. For alkaloid content (Figure 5E,F), under CK treatment, Strain 1 exhibited higher alkaloid levels in both stems and leaves than Strain 2. Following Cd treatment, alkaloid content in stems decreased significantly to low levels in both strains, whereas alkaloid content in leaves showed a marked decrease mainly in Strain 1, with no substantial change observed in Strain 2. In terms of anthocyanin content (Figure 5G,H), under CK treatment, Strain 2 exhibited significantly higher anthocyanin levels in both stems and leaves than Strain 1. After Cd treatment, anthocyanin content in Strain 2 decreased markedly in both tissues, whereas Strain 1 showed relatively minor changes, suggesting a stronger inhibitory effect of Cd on antioxidant pigment synthesis in Strain 2. Regarding soluble protein content (Figure 5I,J), soluble protein levels in both stems and leaves of Strain 2 increased significantly under Cd treatment, whereas Strain 1 showed a decreasing trend. This suggests that Strain 2 may respond to Cd treatment through enhanced synthesis and accumulation of related proteins. Collectively, these results indicate that the two strains showed distinct adaptive responses under Cd treatment through differential regulation of bioactive constituent contents and primary metabolite accumulation.

### 2.5. Rhizosphere Microbial Community Structure and Functional Responses Under Cd Treatment

#### 2.5.1. Rhizosphere Microbial Community Diversity Analysis

**Figure 6:**
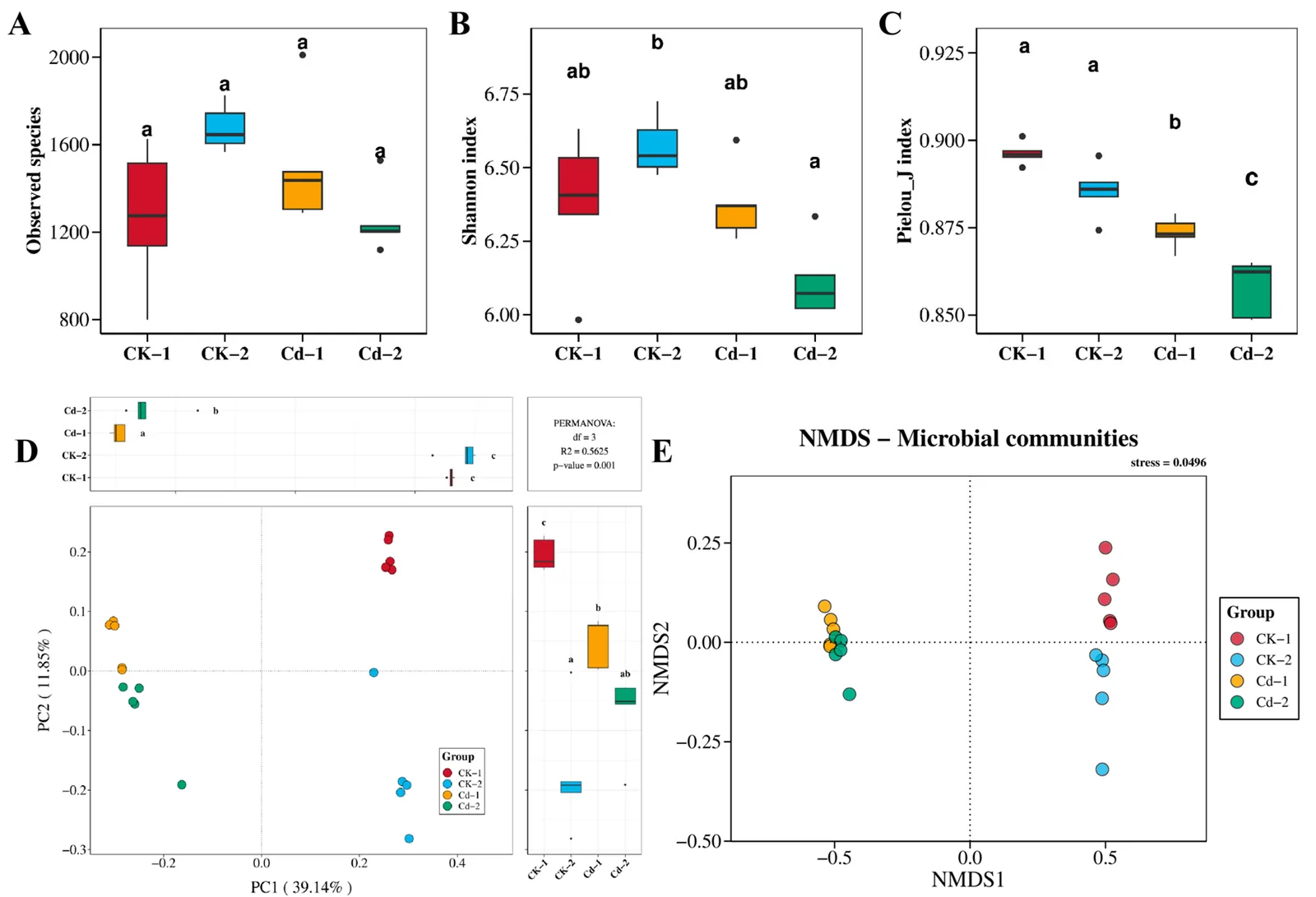
Changes in rhizosphere microbial community diversity and structure of different *D. officinale* strains under different treatments. (**A**) Observed species index. (**B**) Shannon index. (**C**) Pielou evenness index. (**D**) PCoA plot. (**E**) NMDS plot. After Cd treatment, no significant differences in species richness were observed between rhizosphere microbial communities of the two strains, whereas community diversity and evenness were reduced. The PCoA and NMDS plots based on Bray–Curtis distances showed clear separation among samples under different treatments, with separation primarily associated with Cd treatment. Data are presented as mean ± SD (*n* = 5). Different lowercase letters indicate significant differences among treatments (*p* < 0.05). Strains are numbered as follows: CK-1: Strain 1 under CK treatment; CK-2: Strain 2 under CK treatment; Cd-1: Strain 1 under Cd treatment; Cd-2: Strain 2 under Cd treatment.

Alpha diversity analysis showed no significant differences in species richness (observed species) of rhizosphere microbial communities among different Cd treatments and strains (Figure 6A). However, both the Shannon index and the Pielou evenness index exhibited significant differences between treatments, with Cd treatment groups exhibiting lower values than CK treatment groups (Figure 6B,C). These findings indicate that Cd mainly influences rhizosphere microbial community diversity through changes in community evenness rather than reductions in species richness.

Principal coordinate analysis (PCoA) based on Bray–Curtis distances (Figure 6D) showed clear separation among CK-1, CK-2, Cd-1, and Cd-2 samples in ordination space, indicating significant effects of different treatments on rhizosphere microbial community structure. Non-metric multidimensional scaling (NMDS) analysis (Figure 6E) similarly showed distinct clustering of samples from each treatment, with a low stress value (stress = 0.0496), supporting the reliability of the ordination and confirming the consistency with PCoA results. Collectively, the PCoA and NMDS results indicated that samples were primarily separated according to Cd treatment (CK vs. Cd), followed by clustering according to strain under the same treatment conditions. Permutational multivariate analysis of variance (PERMANOVA) further validated these observations, with treatment explaining a substantial proportion of the variation in microbial community structure (R^2^ = 0.5625, *p* = 0.001). These results indicate that Cd treatment was the main factor associated with changes in the rhizosphere microbial community structure of *D. officinale*, while strain effects were relatively weaker.

#### 2.5.2. Rhizosphere Microbial Community Composition Characteristics

**Figure 7:**
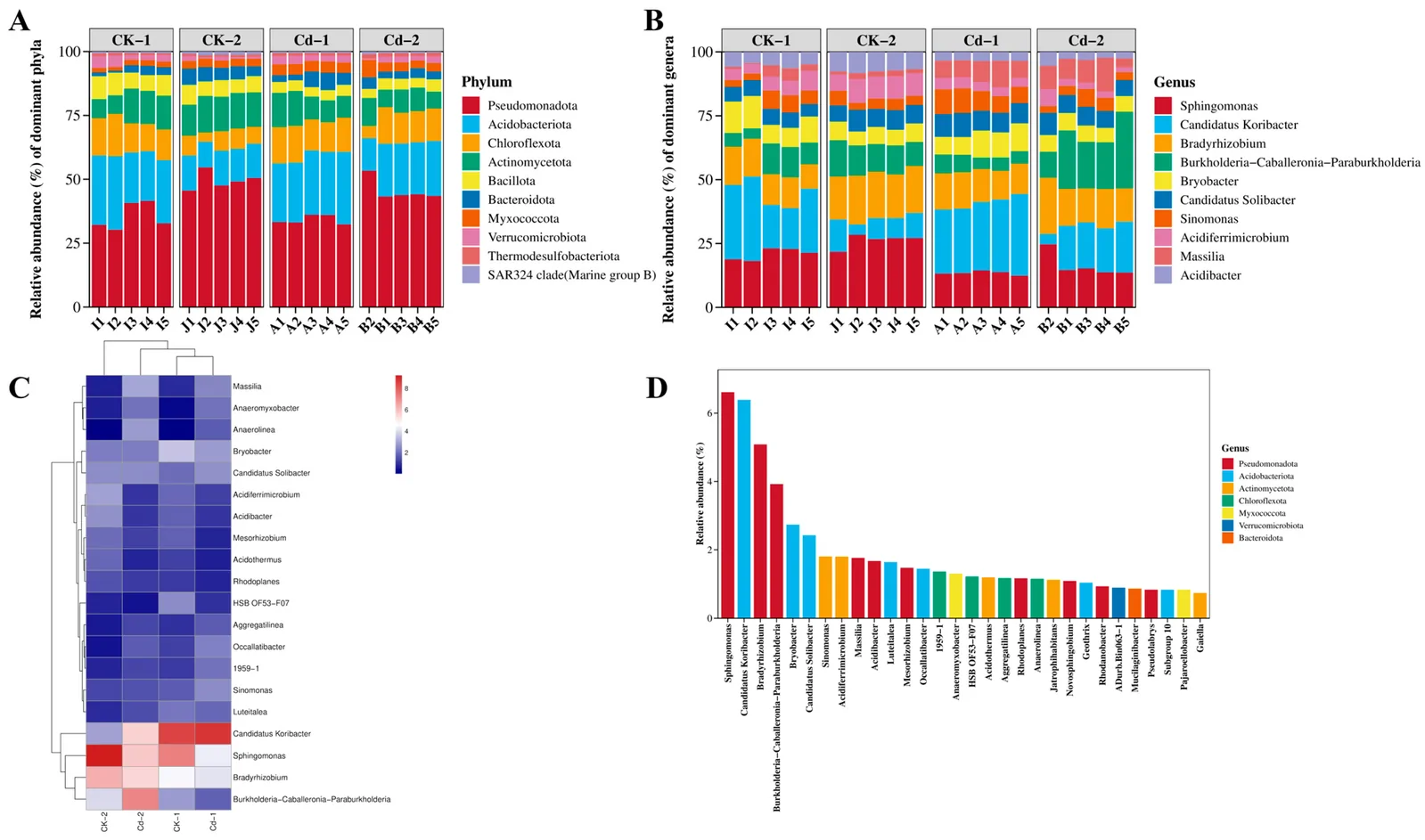
Changes in rhizosphere microbial community composition and structure of different *D. officinale* strains under different treatments. (**A**) Community composition at the phylum level. (**B**) Community composition at the genus level. (**C**) Heatmap of dominant genera at the genus level. (**D**) Bar chart of dominant genera at the genus level. Cd treatment altered the composition of the rhizosphere microbial community, with Strain 2 showing greater shifts in community structure under Cd treatment. Data are presented as mean ± SD (*n* = 5). Strains are numbered as follows: CK-1: Strain 1 under CK treatment; CK-2: Strain 2 under CK treatment; Cd-1: Strain 1 under Cd treatment; Cd-2: Strain 2 under Cd treatment.

At the phylum level, stacked bar charts (Figure 7A) revealed that the rhizosphere microbial communities in all treatment groups were dominated by Pseudomonadota, Acidobacteriota, Chloroflexota, and Actinomycetota. Under CK treatment, the relative abundance of Pseudomonadota was generally higher in Strain 2 than in Strain 1, whereas Strain 1 exhibited a higher proportion of Acidobacteriota. Following Cd treatment, Pseudomonadota decreased in both strains, with a more pronounced decline observed in Strain 2. Concurrently, Strain 2 showed a more marked increase in the relative abundances of Acidobacteriota and Chloroflexota, indicating a stronger shift in community structure under Cd treatment. Nevertheless, the overall composition of dominant phyla remained consistent across treatments, suggesting that Cd treatment mainly affected the relative abundances of dominant phyla while maintaining the overall community framework.

At the genus level, stacked bar charts further revealed differences in microbial community composition among treatment groups. Figure 7B shows that the dominant genera across all treatment groups included *Sphingomonas*, *Candidatus Koribacter*, and *Bradyrhizobium*. Under CK treatment, the relative abundances of *Sphingomonas* and *Bradyrhizobium* were generally higher in Strain 2 than in Strain 1, whereas Strain 1 exhibited a higher proportion of *Candidatus Koribacter*. Following Cd treatment, *Candidatus Koribacter* showed an enrichment trend in both strains, with a more pronounced increase observed in Strain 1. Meanwhile, the relative abundance of the *Burkholderia-Caballeronia-Paraburkholderia* group increased substantially in Strain 2 under Cd treatment, resulting in a more dispersed compositional profile of the community structure in this strain. These observations indicate that Strain 2 showed more pronounced changes in community composition at the genus level under Cd treatment.

To further compare the distribution characteristics of dominant genera among treatments, intergroup heatmap analysis was performed on the major dominant genera (Figure 7C). Samples from CK and Cd treatment formed distinct clusters in the hierarchical clustering analysis, indicating that Cd treatment significantly altered the rhizosphere microbial community structure. Among the dominant genera, the relative abundances of *Sphingomonas* and *Bradyrhizobium* were higher in Strain 2 than in Strain 1, whereas *Candidatus Koribacter* showed markedly higher relative abundance in Strain 1 compared with Strain 2, suggesting strain-associated distribution patterns of dominant microbial genera. Furthermore, a bar chart showing the relative abundances of dominant genera at the genus level was used to compare community composition across treatments (Figure 7D). The results indicated that community differences were primarily driven by a few dominant genera, with *Sphingomonas*, *Candidatus Koribacter*, and *Bradyrhizobium* contributing substantially to intergroup variation. Considering strain differences, Strain 2 showed greater changes in the relative abundances of genera such as *Sphingomonas* and *Bradyrhizobium* under Cd treatment, whereas Strain 1 was characterized by significant enrichment of *Candidatus Koribacter* and other genera. These dominant genera exhibited marked abundance differences between CK and Cd treatments, suggesting that Cd treatment influenced overall community structure by modifying the relative abundances of key dominant genera.

#### 2.5.3. Analysis of Core and Differential Rhizosphere Microbial Community Taxa

**Figure 8:**
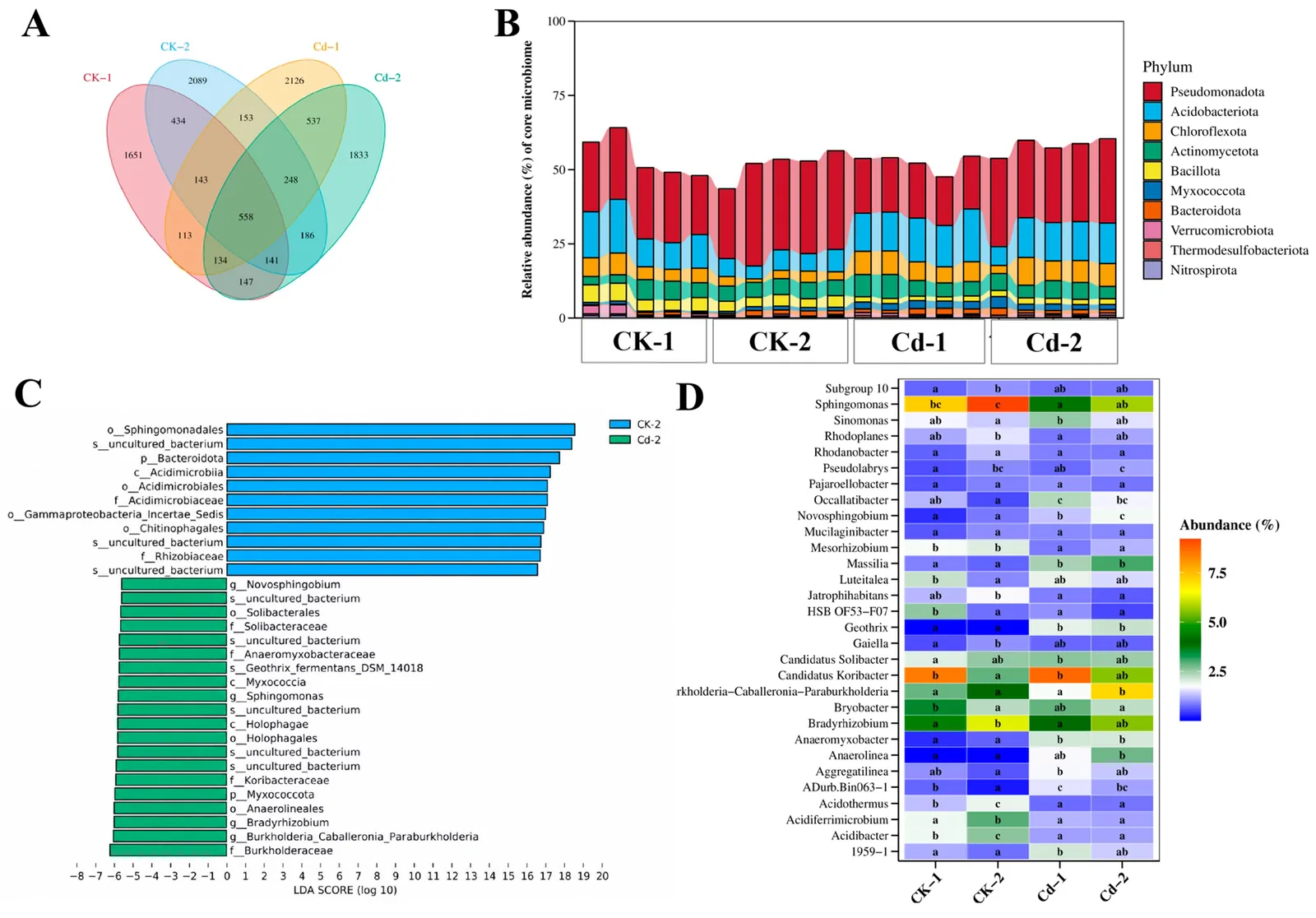
Analysis of core rhizosphere microbial community composition of different *D. officinale* strains under different treatments. (**A**) Venn diagram of core ASVs among groups. (**B**) Relative abundance of core ASVs at the phylum level across samples. (**C**) LEfSe bar plot of LDA score distribution. (**D**) Heatmap of differential genera at the genus level among groups. Under CK treatment, the rhizosphere microbial community of Strain 2 was characterized by discriminatory taxa including Sphingomonadales, Acidimicrobiales, and Chitinophagales. Under Cd treatment, Strain 2 showed enrichment of taxa including *Sphingomonas*, *Bradyrhizobium*, and *Novosphingobium*. Data are presented as mean ± SD (*n* = 5). Different lowercase letters indicate significant differences between treatments (*p* < 0.05). Strains are numbered as follows: CK-1: Strain 1 under CK treatment; CK-2: Strain 2 under CK treatment; Cd-1: Strain 1 under Cd treatment; Cd-2: Strain 2 under Cd treatment.

To characterize the composition of core rhizosphere microbial communities across different strains and treatments, the core amplicon sequence variants (ASVs) of each group were compared. The Venn diagram presented in Figure 8A revealed 558 ASVs shared by all four groups (CK-1, CK-2, Cd-1, and Cd-2), indicating the presence of a common component of the rhizosphere microbial community across treatments. Concurrently, each treatment group possessed a substantial number of unique core ASVs, with CK-1, CK-2, Cd-1, and Cd-2 containing 1651, 2089, 2126, and 1833 unique core ASVs, respectively, demonstrating that Cd treatment and strain were associated with differences in the composition of the core microbial community. Additionally, a certain number of partially shared core ASVs were observed among different groups, suggesting that the core microbial community showed partial variation while maintaining overall stability.

The taxonomic composition of the core ASVs was further analyzed at the phylum level (Figure 8B). The results showed that the core microbial community was dominated by Pseudomonadota, Acidobacteriota, Chloroflexota, Actinomycetota, and Bacillota. The overall composition of dominant phyla was generally consistent across samples, although the relative abundances of specific phyla varied to some extent among samples. Overall, these results indicate that the core microbial community maintained a similar compositional structure at the phylum level across different treatments and strains, suggesting that the core ASVs were relatively stable within the rhizosphere microbial community. A comparison of differential microbial taxa among treatment groups was performed using Linear Discriminant Analysis Effect Size (LEfSe) analysis, and the abundance changes of major differential genera were further illustrated using a genus-level intergroup heatmap. The results revealed that each treatment group was characterized by distinct discriminatory taxa. The LEfSe bar plot of LDA scores (LDA ≥ 5.5; Figure 8C) showed that, under CK treatment, Strain 2 exhibited significantly enriched taxa, primarily within Bacteroidota, Acidimicrobiia, and their associated orders and families, such as Sphingomonadales, Acidimicrobiales, and Acidimicrobiaceae, suggesting that community differences under CK treatment were mainly reflected at higher taxonomic levels. In contrast, under Cd treatment, Strain 2 displayed distinct differential taxa, with discriminatory taxa more concentrated at the genus and species levels, including *Sphingomonas*, *Bradyrhizobium*, *Novosphingobium*, and *Geothrix fermentans*. The genus-level heatmap (Figure 8D) further supported these patterns, showing that genera such as *Bradyrhizobium*, *Geothrix*, *Burkholderia-Caballeronia-Paraburkholderia*, and *Novosphingobium* generally exhibited higher relative abundances under Cd treatment. Collectively, different treatment groups showed distinct microbial community compositions through the enrichment of different dominant taxa, providing evidence for associations between microbial community shifts and Cd treatment.

#### 2.5.4. Functional Prediction Analysis of Rhizosphere Microbial Community

**Figure 9:**
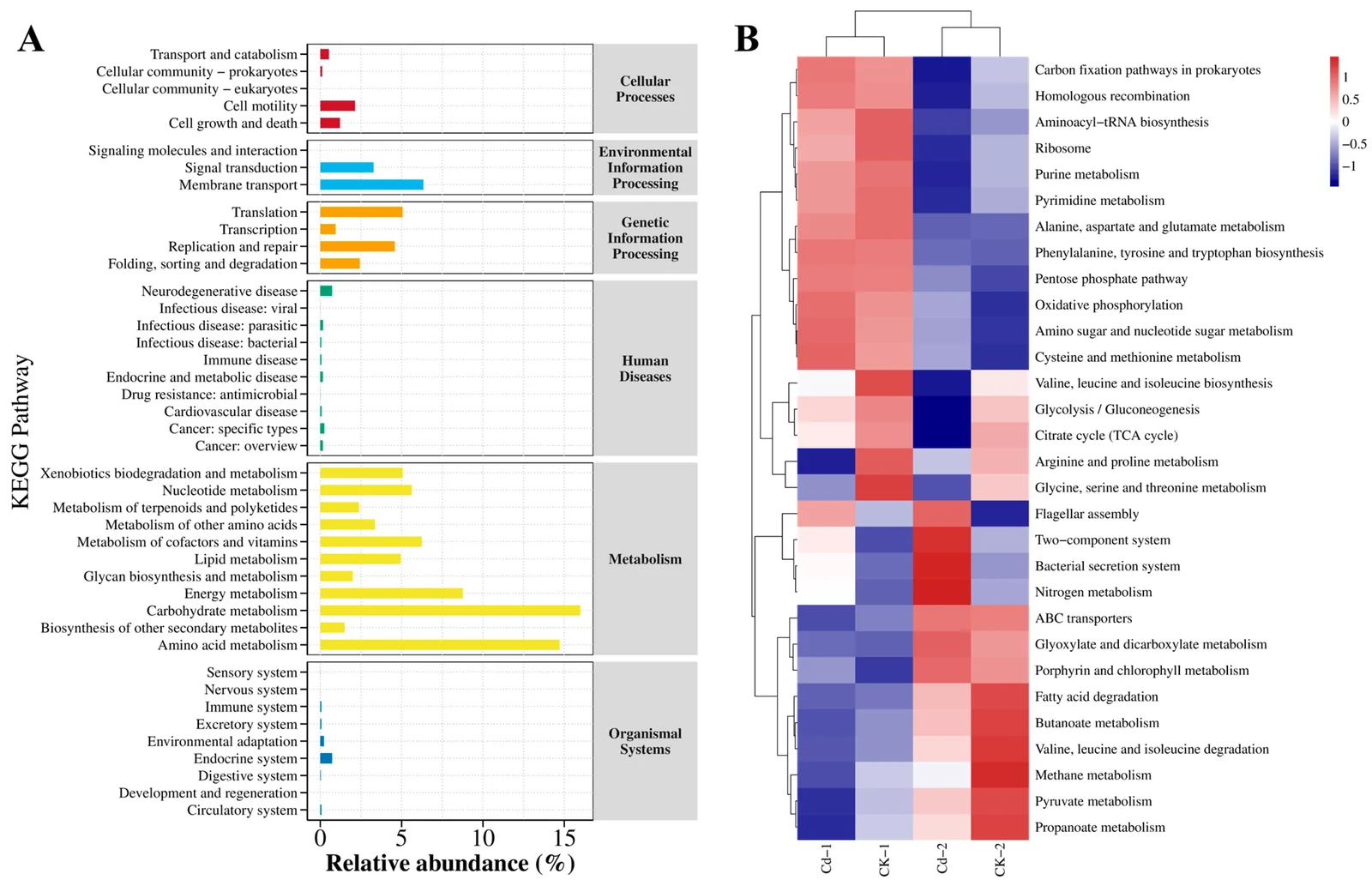
Functional prediction analysis of rhizosphere microbial community of different *D. officinale* strains under different treatments. (**A**) KEGG pathway abundance statistics. (**B**) Level-3 PICRUSt functional annotation clustering heatmap. Under Cd treatment, the rhizosphere microbial communities of Strain 1 showed enrichment of functions associated with energy metabolism and cellular maintenance, whereas those of Strain 2 showed greater enrichment of environmental sensing and detoxification-associated functions. These findings indicate different functional response patterns of rhizosphere microbial communities associated with different *D. officinale* strains under Cd treatment. Data are presented as mean ± SD (*n* = 5). Strains are numbered as follows: CK-1: Strain 1 under CK treatment; CK-2: Strain 2 under CK treatment; Cd-1: Strain 1 under Cd treatment; Cd-2: Strain 2 under Cd treatment.

KEGG-based functional prediction results (Figure 9A) revealed that the predicted functions of the rhizosphere microbial communities were predominantly associated with metabolism-related pathways, with metabolic functions representing the predominant functional category in the Level-1 functional classification. At the Level-2 functional hierarchy, carbohydrate metabolism, amino acid metabolism, and energy metabolism pathways exhibited relatively high abundances, representing major components of the predicted functional profiles of the microbial communities. These findings indicate that the rhizosphere microbial community in this study system showed high predicted metabolic potential for material transformation and energy acquisition.

Further analysis using a Level-3 functional annotation clustering heatmap (Figure 9B) revealed distinct differences in predicted functional profiles among treatment groups. Under CK treatment, the rhizosphere microbial communities of Strain 1 were enriched in pathways including the citrate cycle (TCA cycle), glycolysis/gluconeogenesis, arginine and proline metabolism, and glycine, serine, and threonine metabolism, suggesting enrichment of pathways associated with anabolic metabolism and cellular biosynthesis under non-stress conditions. In contrast, the rhizosphere microbial communities of Strain 2 showed higher relative abundances of pathways including fatty acid degradation, valine, leucine and isoleucine degradation, and pyruvate metabolism, indicating a functional profile characterized by enhanced carbon utilization and energy metabolism. Cd treatment altered these predicted functional profiles, with the two strains displaying different functional responses. Under Cd treatment, the rhizosphere microbial communities of Strain 1 showed increased relative abundances of pathways associated with energy metabolism and cellular maintenance, including oxidative phosphorylation, carbon fixation pathways in prokaryotes, and homologous recombination. The enrichment of oxidative phosphorylation and carbon fixation pathways suggests changes associated with energy metabolism adjustment under Cd treatment, whereas the increased abundance of homologous recombination may be associated with DNA repair processes related to metal-induced oxidative stress. In contrast, under Cd treatment, the rhizosphere microbial communities of Strain 2 were enriched in pathways associated with environmental sensing and detoxification-related functions. These included the two-component system, which is involved in bacterial perception and response to environmental signals such as heavy metals; the bacterial secretion system, which is associated with extracellular substance transport and metabolite exchange; and nitrogen metabolism, which may be related to the synthesis of nitrogen-containing compounds involved in stress responses. Collectively, these functional differences indicate that the rhizosphere microbial communities of Strain 1 under Cd treatment were characterized by enhanced energy metabolism and cellular maintenance-related functions, whereas those of Strain 2 exhibited greater enrichment of environmental sensing and detoxification-associated functions. These findings reveal distinct functional response patterns of the rhizosphere microbial communities associated with different *D. officinale* strains under Cd treatment.

## 3. Discussion

### 3.1. Growth Responses and Cd Accumulation Patterns

For medicinal plants, exposure to heavy metal-contaminated environments commonly causes growth inhibition and developmental alterations in roots and aboveground tissues [33]. These phenotypic responses reflect plant tolerance strategies and directly affect heavy metal uptake and distribution within plants [34]. Therefore, understanding the relationship between growth performance and heavy metal accumulation is essential for evaluating stress adaptation as well as medicinal safety and cultivation risks.

In the present study, distinct *D. officinale* strains exhibited contrasting responses to Cd treatment. Strain 1 showed the strongest growth inhibition but maintained relatively low Cd concentrations in roots, stems, and leaves, whereas Strain 2 maintained comparatively stable growth while accumulating substantially higher Cd levels in all tissues. These results indicate that growth tolerance and Cd accumulation capacity are not necessarily coupled in *D. officinale*, and that enhanced growth stability does not always correspond to reduced Cd accumulation risk.

Such divergence may arise from strain-specific regulation of Cd uptake, root-to-shoot translocation, intracellular sequestration, and cellular efflux, processes that collectively determine Cd accumulation in aboveground tissues [35]. Therefore, the lower absolute Cd concentrations observed in Strain 1 indicate lower overall Cd accumulation. These interpretations are further supported by the contrasting expression patterns of metal transport-related genes described below.

### 3.2. Antioxidant Regulation and Molecular Responses to Cd Treatment

Oxidative stress regulation represents a key physiological mechanism underlying plant responses to Cd toxicity [36]. Differences in antioxidant capacity influence cellular homeostasis and may subsequently affect metabolic processes associated with medicinal quality formation. In this study, MDA accumulation increased in Strains 2, 3, and 4, whereas it remained nearly unchanged in Strain 1. However, Strain 1 exhibited increased POD and SOD activities, suggesting an effective antioxidant response to alleviate Cd-induced oxidative damage. In contrast, Strain 2 showed elevated MDA accumulation accompanied by reduced CAT and POD activities, indicating impaired antioxidant homeostasis.

Because Cd is not a redox-active metal itself, its oxidative effects are generally associated with disturbance of cellular redox homeostasis rather than direct redox cycling. In this context, changes in SOD, POD, and CAT activities can influence the removal of reactive oxygen species and thereby affect the extent of membrane lipid peroxidation. The coordinated increase in SOD and POD activities in Strain 1 is therefore consistent with a stronger capacity to buffer Cd-induced oxidative stress, whereas the reduced CAT and POD activities in Strain 2 may have limited its ability to maintain ROS homeostasis.

However, the relationship between Cd accumulation and oxidative damage is likely to be bidirectional and complex. Higher internal Cd concentrations may increase cellular stress, while differences in antioxidant capacity may also determine how effectively each strain tolerates a given Cd burden. Thus, the contrasting phenotypes of the two strains are more appropriately interpreted as the combined outcome of metal homeostasis and antioxidant regulation rather than as a simple consequence of Cd concentration alone.

At the molecular level, differential expression of metal transport- and detoxification-related genes further revealed distinct Cd response strategies between strains. In Strain 1, Cd treatment induced sustained expression of *HMA3*, *HMA5*, and *ALMT*, particularly during the early response stage (12–24 h after Cd treatment), which may contribute to Cd sequestration, efflux, and restricted translocation to aboveground tissues. Among these genes, *HMA*-family transporters are closely associated with cellular heavy-metal homeostasis. The increased expression of *HMA3* in Strain 1 is consistent with enhanced intracellular sequestration of Cd into storage compartments, which could reduce the cytosolic Cd pool available for long-distance transport. Increased *HMA5* expression may likewise reflect strengthened metal efflux or redistribution processes. The induction of *ALMT* may indicate an additional adjustment of membrane transport and ion/organic-acid homeostasis under Cd treatment.

In contrast, Strain 2 showed stronger induction of *NRAMP1*, *NRAMP2*, and *ZIP*, genes associated with metal uptake and long-distance transport, consistent with its higher Cd accumulation in stems and leaves. *NRAMP*- and *ZIP*-family transporters generally participate in the transport of divalent metal ions and may show limited substrate specificity. Their differential expression in Strain 2 may be associated with its higher absolute Cd concentrations in different tissues. In particular, simultaneous activation of several metal uptake-related transporters could increase the pool of Cd entering root cells and available for vascular transport, thereby contributing to the higher Cd concentrations measured in stems and leaves.

The response of Strain 1 appears to involve a combination of enhanced antioxidant protection and transcriptional regulation favoring Cd sequestration or restricted movement, whereas Strain 2 combines relatively stable growth with stronger expression of genes associated with metal uptake and transport. This coordinated physiological and transcriptional divergence provides a mechanistic framework for understanding why the two strains differ in Cd accumulation despite both being able to survive under Cd exposure.

### 3.3. Changes in Bioactive Constituents Under Cd Treatment

Cd treatment also altered the accumulation profiles of bioactive constituents in *D. officinale*. Cd exposure generally reduced polysaccharide, flavonoid, and alkaloid contents, although the extent of reduction varied between strains and tissues [37]. Compared with Strain 1, Strain 2 maintained relatively higher levels and smaller fluctuations of several bioactive constituents, indicating greater metabolic stability under Cd treatment. In particular, the increased soluble protein content in Strain 2 suggests that enhanced protein metabolism may contribute to stress adaptation.

The reduction in major bioactive constituents may reflect a redistribution of metabolic resources under Cd treatment. When plants are exposed to heavy metals, resources required for normal growth and secondary metabolite biosynthesis may also be used to maintain cellular homeostasis, repair stress-induced damage, and support detoxification processes [38]. Therefore, changes in polysaccharides, flavonoids, and alkaloids may represent part of a broader metabolic adjustment rather than isolated alterations in individual compounds.

The relatively stable bioactive constituent profiles observed in Strain 2, despite its higher Cd accumulation, suggest that this strain may maintain certain metabolic processes under Cd exposure more effectively than Strain 1. However, higher metabolic stability should not be equated with greater medicinal safety because Strain 2 also accumulated more Cd in its tissues. Thus, evaluation of *D. officinale* germplasm under heavy-metal stress should consider both the maintenance of bioactive constituents and the accumulation of toxic elements.

### 3.4. Rhizosphere Microbial Community Responses to Cd Treatment

Rhizosphere microbial communities represent an integral component of plant stress adaptation, as their community structure and functional potential contribute to nutrient acquisition and environmental responses [39]. Cd treatment significantly reshaped the rhizosphere microbial communities associated with *D. officinale*. Alpha diversity analysis indicated that Cd mainly reduced microbial community evenness rather than species richness, with stronger effects observed in Strain 2. Beta diversity analysis further demonstrated that Cd concentration was the primary factor determining microbial community variation, whereas strain identity exerted a secondary influence. Community composition and LEfSe analyses revealed Cd-induced enrichment of potentially metal-responsive taxa, including *Sphingomonas*, *Bradyrhizobium*, the *Burkholderia-Caballeronia-Paraburkholderia* group, and *Candidatus Koribacter*. These microbial groups are associated with organic matter transformation, metal interaction, and environmental adaptation. Their enrichment under Cd treatment may therefore represent a community-level response to the altered rhizosphere environment.

Under CK treatment, taxa related to nutrient cycling and rhizosphere stability, such as Acidiferrimicrobium and Acidibacter, were relatively enriched, potentially supporting normal plant growth. The transition from these CK-associated taxa toward a community enriched in putatively stress-responsive microorganisms suggests that Cd exposure changes the ecological filtering conditions within the rhizosphere. Such filtering may arise from direct Cd toxicity to microorganisms as well as indirect changes in the root-associated environment caused by plant stress responses.

Thus, Cd-induced microbial restructuring may provide an ecological buffer by modulating metabolic functions, energy acquisition, and material transformation within the rhizosphere. Nevertheless, because the present study primarily characterized microbial community composition rather than experimentally verifying microbial functions, the proposed buffering effect should be considered a plausible ecological interpretation. Future isolation or functional studies of the enriched taxa would be necessary to determine whether they directly influence Cd availability, plant uptake, or stress tolerance.

### 3.5. Integrated Plant-Rhizosphere Adaptation Model Under Cd Treatment

In summary, different *D. officinale* strains employed distinct adaptive strategies under Cd treatment through coordinated regulation of physiological responses, gene expression, bioactive constituent metabolism, and rhizosphere microbial community interactions. Strain 1 exhibited relatively low Cd concentrations in its tissues, together with increased POD and SOD activities, suggesting a potentially effective antioxidant response despite pronounced growth inhibition. In contrast, Strain 2 exhibited higher Cd concentrations in its tissues, accompanied by changes in bioactive constituent accumulation and rhizosphere microbial community composition.

**Figure 10:**
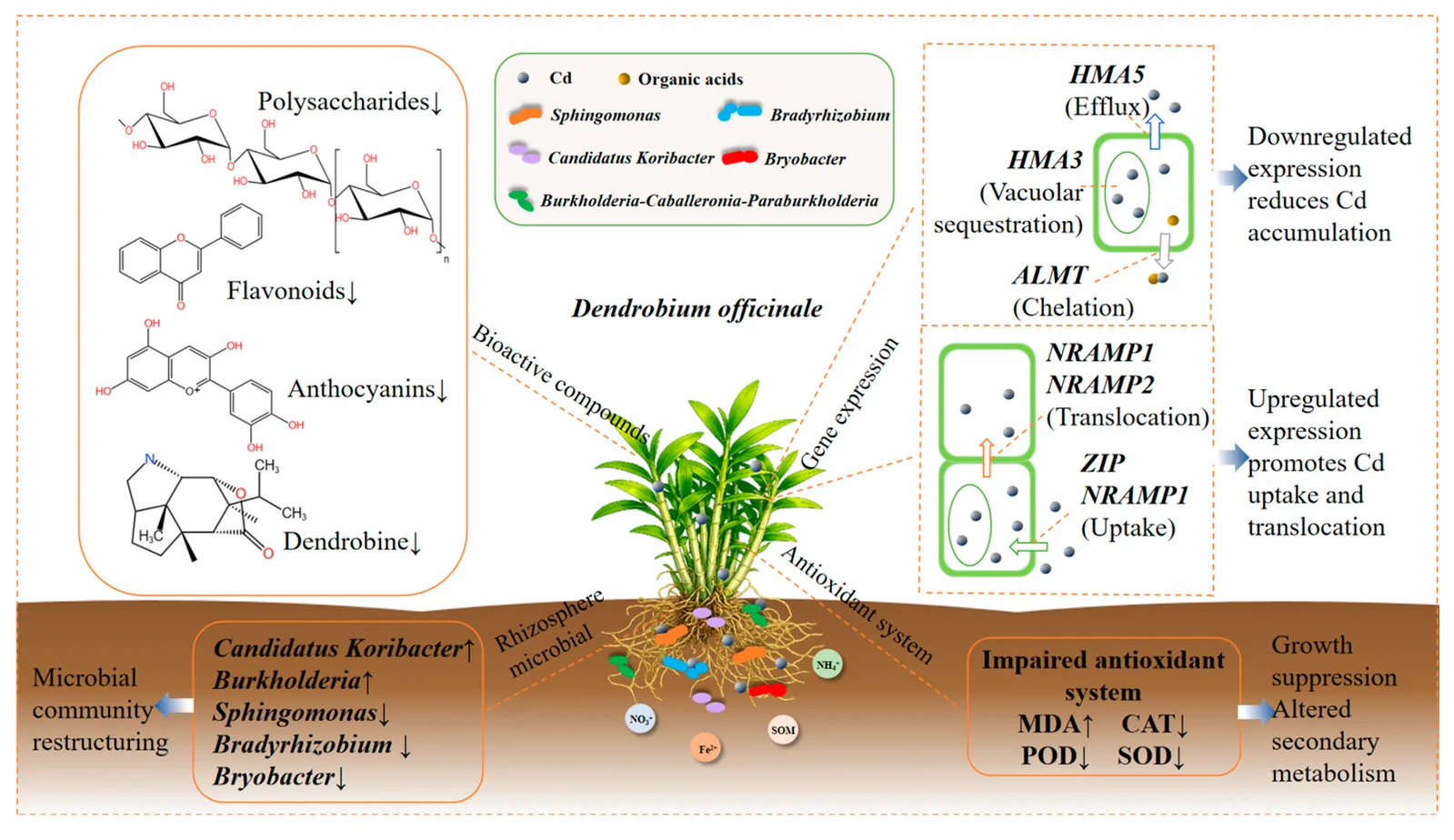
Schematic representation of the coordinated adaptive response mechanism of the Cd-*D. officinale*-rhizosphere microbial community interaction system. The proposed model integrates strain-specific changes in Cd uptake, translocation and sequestration, metal transport-related gene expression, antioxidant responses, bioactive constituent accumulation, and rhizosphere microbial community restructuring under Cd stress.

Based on these findings, a synergistic adaptation model involving interactions between *D. officinale* and its rhizosphere microbial community is proposed (Figure 10). Cd treatment induced strain-specific responses by regulating metal transport, antioxidant metabolism, bioactive constituent accumulation, and microbial community characteristics, ultimately affecting the balance between plant growth, medicinal quality, and safety. From a medicinal safety perspective, Strain 1 is preferable for cultivation in Cd-contaminated soils due to its lower Cd accumulation in medicinal tissues. These findings provide insights into the selection of elite *D. officinale* strains and the development of safe cultivation strategies under heavy metal stress conditions.

## 4. Materials and Methods

### 4.1. Plant Materials and Culture Conditions

The *D. officinale* seedlings used in this study were obtained from the Taizhou *D. officinale* cultivation base. The four strains used here, namely Strain 1 (Taizhou strain), Strain 2 (Xianju strain), Strain 3 (Fujian strain), and Strain 4 (Wenzhou strain), have been previously described in our earlier studies [19]. These strains were maintained separately at the cultivation base and were distinguished according to morphological characteristics. All samples were authenticated by Prof. Dequan Zhang from the College of Life Science and Technology, Southwest University of Science and Technology, and Dendrobium expert Binguo Shen from Fenghuangyuan Biotechnology Co., Ltd. (Taizhou, China). Tissue culture, experimental procedures, and sample storage were conducted at China Jiliang University (Hangzhou, China), following protocols previously established by our research group [19].

Healthy *D. officinale* plantlets were selected, surface sterilized, and cut into stem segments approximately 2 cm long, each containing nodes. These segments were inoculated onto adventitious bud induction medium containing 6.2 g·L^−1^ agar (Shanghai Yuanye Bio-Technology Co., Ltd., Shanghai, China), 30 g·L^−1^ sucrose (Shanghai Yuanye Bio-Technology Co., Ltd., Shanghai, China), 4.43 g·L^−1^ MS basal salts (PhytoTech Labs, Lenexa, KS, USA), 100 mL·L^−1^ coconut milk, 2 mg·L^−1^ 6-benzylaminopurine (6-BA, Macklin, Shanghai, China), and 1 mg·L^−1^ 1-naphthaleneacetic acid (NAA, Macklin, Shanghai, China) [40]. Each flask contained 4–6 stem segments and was incubated at 25 °C under a 12 h·d^−1^ photoperiod. Shoot primordia developed 15 days after inoculation. After 4 weeks, healthy adventitious shoots with uniform morphology and approximately 1.5 cm in height were selected and transferred to rooting medium containing 6.2 g·L^−1^ agar, 30 g·L^−1^ sucrose, 3.21 g·L^−1^ B5 basal salts (PhytoTech Labs, Lenexa, KS, USA), 100 mL·L^−1^ coconut milk, 0.2 mg·L^−1^ indole-3-butyric acid (IBA, Macklin, Shanghai, China), and 0.5 mg·L^−1^ NAA [41]. Each flask contained 4–6 adventitious shoots. The procedure was repeated until all explants had been successfully subcultured.

### 4.2. Soil Cd Treatment and Transplanting of Plantlets

The experimental soil was acidic red soil collected from Linggen Village, Taizhou City, Zhejiang Province, China. Before the experiment, the soil was air-dried, ground, sieved to remove stones and visible plant residues, and stored until use. The initial physicochemical properties of the soil were as follows: pH 5.32, organic matter 11.252 g·kg^−1^, total nitrogen 0.526 g·kg^−1^, total phosphorus 0.303 g·kg^−1^, total potassium 4.541 g·kg^−1^, available nitrogen 59.645 mg·kg^−1^, available phosphorus 1.520 mg·kg^−1^, and available potassium 118.079 mg·kg^−1^. Before the formal experiment, a preliminary trial was conducted to evaluate the suitability of red soil as a cultivation substrate for *D. officinale*. The results showed that *D. officinale* exhibited normal growth without apparent stress symptoms, indicating that the substrate was suitable for subsequent experiments. Accordingly, red soil was selected for all subsequent analyses. Two Cd concentration levels (0 and 0.9 mg·kg^−1^) were established using a CdCl_2_ (Shanghai Aladdin Biochemical Technology Co., Ltd., Shanghai, China) solution as the Cd source. Each container was filled with 5 kg of pretreated red soil, followed by the gradual addition of the corresponding CdCl_2_ solution. The soil was then thoroughly mixed to ensure homogeneity. During the following 60 days, the soil was thoroughly mixed once per week, and water was added as needed to maintain soil moisture at approximately 65% of field capacity to ensure a stable and uniform distribution of Cd [42].

The experiment was designed as a two-factor factorial experiment involving four *D. officinale* strains (strain 1, strain 2, strain 3, and strain 4) and two Cd concentration levels (0 and 0.9 mg·kg^−1^). For each strain × Cd treatment combination, 50 pots were established, with 4–6 plants per pot, giving a total of 400 pots. For Cd concentration determination, three biological replicates were analyzed for each treatment.

Healthy *D. officinale* plantlets of uniform size (6 months old) were selected for the experiment. Before transplanting, plantlets of the four strains were acclimated for 2–3 weeks to allow a gradual transition from the closed, stable tissue culture environment to natural growth conditions and to facilitate adaptation [43]. After removal from the culture flasks, the roots were rinsed thoroughly to remove residual culture medium, washed three times with double-distilled water (ddH_2_O), and air-dried until the roots turned off-white to improve transplant survival. The transplanting substrate consisted of a mixture of red soil and pine bark at a ratio of 2:1 (*v*/*v*) [44]. Plantlets of the four *D. officinale* strains were transplanted separately into the mixed substrate containing the two Cd concentration treatments and grown under identical conditions. All transplanted plants were cultivated in a greenhouse (25 °C, 12 h·d^−1^ photoperiod) and irrigated once per week to maintain sufficient moisture for normal growth.

### 4.3. Sampling, Growth Trait Measurement, and Cd Content Determination

Samples were collected 1 month after Cd treatment. To remove surface-bound Cd, the roots of *D. officinale* plantlets were immersed in EDTA (Hangzhou Mike Chemical Instrument Co., Ltd., Hangzhou, China) solution for 20 min and then thoroughly rinsed with ddH_2_O.

To evaluate the effects of Cd treatment on growth, growth traits were measured for each strain 1 month after treatment. For each strain, ten healthy plants of uniform size were randomly selected as biological replicates. The following growth traits were measured: root length (maximum primary root length measured with a ruler), stem length (distance from the stem base to the shoot apex), stem diameter (measured 1 cm above the root base with a vernier caliper), and fresh weight (fresh weight of the whole plant measured with an electronic balance). All measurements were performed under identical environmental conditions and completed immediately after sampling to minimize the effects of water transpiration on the measurements. The resulting data were used to compare differences in growth traits among different strains and across different Cd treatment levels.

Plants were separated into their roots, stems, and leaves. Fresh tissue subsamples were immediately flash-frozen in liquid nitrogen and stored at −80 °C to preserve physiological characteristics and RNA integrity for subsequent physiological analyses and gene expression analyses. For Cd content determination, root, stem, and leaf samples were placed in an oven at 105 °C for 2 h to inactivate enzymatic activity, followed by drying at 70 °C to constant weight. Dried samples were ground and digested using a microwave digestion system. Cd content was determined using graphite furnace atomic absorption spectrophotometry (GFAAS) according to the Chinese national standard Determination of Cadmium in Foods (GB 5009.15-2014) [45]. Separate samples were dried at 50 °C to constant weight for the determination of polysaccharides, flavonoids, and total alkaloids. Fresh stem and leaf tissues were used for the determination of anthocyanin and soluble protein. Anthocyanin content was expressed as µg·g^−1^ FW, whereas soluble protein was reported as the concentration in the tissue extract (g·L^−1^). Soil samples were oven-dried, crushed, and passed through a 200-mesh sieve. The sieved samples were digested using a microwave digestion system, and Cd content in the digestion solution was determined according to the Chinese national standard Soil Quality−Determination of Lead and Cadmium−Graphite Furnace Atomic Absorption Spectrophotometry (GB/T 17141-1997) [46].

### 4.4. Physiological Characteristic Measurement and Cd-Related Gene Expression Analysis

MDA content and the CAT, POD, and SOD in leaves were determined using commercial assay kits (Nanjing Jiancheng Bioengineering Institute, Nanjing, China). The following kits were used: MDA assay kit (A003-1-1), CAT assay kit (A007-1-1), POD assay kit (A084-3-1), and total SOD (T-SOD) assay kit (A001-1). All assays were performed according to the manufacturer’s instructions. Three biological replicates were analyzed for each treatment.

To investigate the molecular basis of the differential responses among distinct *D. officinale* strains, homology searches were performed against the NCBI database using sequences of well-characterized heavy metal-related genes from other plant species (e.g., *OsHMA3*, *OsHMA5*, *AtNramp1*, *AtNramp2*, *ALMT*, and *ZIP*). Homologous genes showing >90% sequence identity, query coverage of ≥80%, and an E-value of ≤1 × 10^−10^ were identified in *D. officinale*, and gene-specific primers were designed accordingly for qRT-PCR. Total RNA was extracted from the roots, stems, and leaves of *D. officinale* using TRIzol reagent (Invitrogen, Carlsbad, CA, USA). Reverse transcription was performed using the PrimeScript™ RT Reagent Kit with gDNA Eraser (Takara Bio Inc., Kusatsu, Shiga, Japan) according to the manufacturer’s instructions to obtain cDNA, which was stored at −20 °C until analysis. qRT-PCR was performed using the TB Green^®^ Premix Ex Taq™ II (Tli RNaseH Plus) kit (Takara Bio Inc., Kusatsu, Shiga, Japan) on a Rotor-Gene Q real-time PCR system (QIAGEN, Hilden, Germany). Reaction mixtures and thermal cycling conditions followed the manufacturer’s instructions. Primer sequences, expected amplicon sizes, PCR amplification efficiencies, and GenBank accession numbers are provided in Supplementary Table S1, while the PCR amplification program is provided in Supplementary Table S2. Three biological replicates were analyzed for each sample, and β-actin was used as the internal reference gene.

### 4.5. Determination of Bioactive Compound Contents

The polysaccharide content of *D. officinale* was determined according to the *Chinese Pharmacopoeia* (2020 edition, Part I) [47]. A 0.3 g sample of powdered *D. officinale* was extracted with 200 mL of ddH_2_O under reflux for 2 h. The extract was processed according to the Pharmacopoeia protocol, and polysaccharide content was determined using the phenol-sulfuric acid method at 488 nm with glucose (Hangzhou Mike Chemical Instrument Co., Ltd., Hangzhou, China) as the standard.

Total flavonoid content was determined according to Li et al. [48]. A 0.3 g sample of powdered *D. officinale* was extracted with 70% ethanol (Hangzhou Mike Chemical Instrument Co., Ltd., Hangzhou, China) under reflux at 80 °C for 2 h. Total flavonoid content was determined using the NaNO_2_-Al(NO_3_)_3_ (Hangzhou Mike Chemical Instrument Co., Ltd., Hangzhou, China)colorimetric method at 510 nm with rutin (Mansite (Chengdu) Biotechnology Co., Ltd., Chengdu, China) as the standard.

Alkaloid content was determined according to Li et al. [48]. A 0.5 g sample of powdered *D. officinale* was pretreated with ammonia solution (Hangzhou Mike Chemical Instrument Co., Ltd., Hangzhou, China) and extracted with chloroform (Hangzhou Mike Chemical Instrument Co., Ltd., Hangzhou, China) under reflux for 2 h. Alkaloid content was determined using the bromocresol green colorimetric method at 620 nm with dendrobine (Mansite (Chengdu) Biotechnology Co., Ltd., Chengdu, China) as the standard.

Anthocyanin content was determined using a commercial total anthocyanin assay kit (ADS-W-KY016-96; Jiangsu Addison Biotechnology Co., Ltd., Nanjing, China). Soluble protein content was determined using a commercial protein quantification (TP) assay kit (A045-2; Nanjing Jiancheng Bioengineering Institute, Nanjing, China). All assays were performed according to the manufacturers’ instructions. Three biological replicates were analyzed for each treatment.

### 4.6. Rhizosphere Microbial Community Analysis

Rhizosphere soil samples were collected from *D. officinale* at the 0–5 cm soil layer adjacent to the roots. After collection, the samples were homogenized, immediately frozen in liquid nitrogen, and stored at −80 °C until analysis. The samples were submitted to a commercial sequencing service provider for microbial community analysis. Bacterial community composition and diversity were analyzed by sequencing the V3–V4 hypervariable region of the 16S rRNA gene amplified using primers 341F (5′-CCTAYGGGRBGCASCAG-3′) and 806R (5′-GGACTACNNGGGTATCTAAT-3′) and subjected to Illumina paired-end 250-bp (PE250) sequencing. Raw sequencing data were subjected to demultiplexing, quality filtering, primer removal, read merging, and chimera removal, after which the valid sequences were imported into QIIME2 (v2020.11.1). DADA2 (v1.18.0) was used with default parameters for sequence denoising and amplicon sequence variant (ASV) inference. A total of 50,012–64,530 valid sequences were obtained per sample, with mean sequence lengths of 410.27–414.99 bp. Taxonomic annotation was performed to obtain community composition, relative abundance, and diversity indices. Microbial functional profiles were predicted using PICRUSt2 (v2.4.1) implemented in QIIME2 with default parameters. Five biological replicates were included for each treatment.

### 4.7. Statistical Analysis

Statistical analyses were performed using IBM SPSS Statistics (v20.0). Differences among treatments were evaluated by two-way analysis of variance (ANOVA), followed by Duncan’s multiple range test, with *p* < 0.05 considered statistically significant. All data are expressed as the mean ± standard deviation (SD). Relative gene expression levels were calculated using the 2^−ΔΔCt^ method. GraphPad Prism (v8.0.2.263) was used for data visualization and figure preparation. Microbial community diversity, composition, differential taxa, and functional prediction were analyzed using the corresponding bioinformatics pipelines and statistical methods described in Section 4.6.

## Figures and Tables

**Table 1:** BCF and TF of Cd in different *D. officinale* strains.

Factor/Strain	1	2	3	4
BCF	1.005	2.342	1.368	1.803
TF	0.656	0.433	0.370	0.644

BCF represents the bioconcentration factor of Cd, reflecting the Cd accumulation capacity of the plant, whereas TF represents the translocation factor, reflecting the transfer of Cd from roots to aboveground tissues. 1–4 correspond to 1, Strain 1 (Taizhou strain); 2, Strain 2 (Xianju strain); 3, Strain 3 (Fujian strain); and 4, Strain 4 (Wenzhou strain).

## Data Availability

The original contributions presented in the study are included in the article; further enquiries can be directed to the corresponding author.

## Supplementary Materials

The following supporting information can be downloaded at https://www.mdpi.com/article/10.3390/plants15203187/s1. Table S1. Primer information for qRT-PCR analysis of target genes in *D. officinale*. Table S2. Reaction system for quantitative real-time PCR (qRT-PCR) *.
