# Supplementary material for: Cadmium Exposure Modulates Quality of the Medicinal Plant Dendrobium officinale via Regulation of Gene Expression, Antioxidant Systems, Bioactive Compound Accumulation, and Rhizosphere Microbial Community
Source: Plants (Basel). 2026 Oct 17;15(20):3187. doi: 10.3390/plants15203187 (PMC13708823; doi:10.3390/plants15203187)
Supplement: Supplementary file 1 [file plants-15-03187-s001.zip › plants-4581261-supplementary.pdf]

Table S1. Primer information for qRT-PCR analysis of target genes in *D. officinale*

Primer name	Sequence (5′–3′)	Amplicon size (bp)	PCR efficiency (%)	GenBank accession No.
β-actin-F	TTGTGTTGGATTCTGGTGATGGTGT	171	98.6	XM_028699662.1
β-actin-R	TTTCCCGTTCTGCTGTTGTTGTGAA			
HMA3-F	TCGACTTGCAAGGAGGACAC	127	96.9	XM_020846020.2
HMA3-R	ATCAGCTAATACGGCTGCCC			
HMA5-F	TGGTTCCTGGTGCAAAGGTT	93	101.4	XM_020850404.2
HMA5-R	CAGGTCGTGCTTCTCCAGTT			
Nramp1-F	TTGCTCTGGATCATCCTCGC	116	99.2	XM_020849765.2
Nramp1-R	GCGTATTCTGCTCGGCAATG			
Nramp2-F	TGGCCATTTCTTTTGCGTGG	129	97.7	XM_020837206.2
Nramp2-R	TTACACAACCCACAACCCCC			
ALMT-F	CGCTTTCAAGCTTGTGCAGT	141	102.3	XM_020817880.2
ALMT-R	TATGTTCCAGCCTCGCCAAG			
ZIP-F	GAAGATGCCACAGACCCTCC	196	95.8	XM_020819062.2
ZIP-R	CCTGGTTAACAGCCGGAACA			

Table S2. Reaction system for quantitative real-time PCR (qRT-PCR)*

Reagent	Volume（10 μL）
TBGREEN II	5
Forward Primer-F	0.4
Reverse Primer-R	0.4
cDNA	1
ddH_2O	3.2

*The reaction system (10 μL) was as follows: TBGREEN II (TaKaRa) 5 μL, 0.4 upstream and 0.4 downstream primers each μL, cDNA 1 μL, and ddH2O 3.2 μL. Using the Rotor-Gene Q Series software 2.0.2, the reaction procedure was carried out as follows: 95 ◦C for 1 min, 95 ◦C for 20 s, 60 ◦C for 30 s, 72 ◦C for 20 s, and finally denature at 95 ◦C for 1 min, anneal to 60 ◦C, hold for 1 min, and then rise to 95 ◦C at a rate of 1 ◦C/s to draw a dissolution curve. Three replicates were obtained for each sample.
